# Development and Physicochemical Properties of Low Saturation Alternative Fat for Whipping Cream

**DOI:** 10.3390/molecules26154586

**Published:** 2021-07-29

**Authors:** Jung-Ah Shin, Yea-Jin Hong, Ki-Teak Lee

**Affiliations:** 1 Department of Food Processing and Distribution, Gangneung-Wonju National University, 7 Jukheon-gil, Gangneung 25457, Korea; 2 Maeil Innovation Center (MIC), Maeil Dairies Co., Ltd., 63 Jinwiseo-ro, Jinwi-myeon, Pyeongtaek-si 17714, Korea; hong6619@maeil.com; 3 Department of Food Science and Technology, Chungnam National University, 99 Daehak-ro, Yuseong-gu, Daejeon 34134, Korea; ktlee@cnu.ac.kr

**Keywords:** whipping cream alternative fat, hydrogenated palm kernel oil, enzymatic acyl migration, fat polymorphism, physicochemical properties

## Abstract

We developed an alternative whipping cream fat using shea butter but with low saturation. Enriched stearic-oleic-stearic (SOS) solid fat was obtained from shea butter via solvent fractionation. Acyl migration reactant, which mainly contains asymmetric SSO triacylglycerol (TAG), was prepared through enzymatic acyl migration to obtain the creaming quality derived from the β’-crystal form. Through enzymatic acyl migration, we obtained a 3.4-fold higher content of saturated-saturated-unsaturated (SSU) TAG than saturated-unsaturated-saturated (SUS) TAG. The acyl migration reactant was refined to obtain refined acyl migration reactant (RAMR). An alternative fat product was prepared by blending RAMR and hydrogenated palm kernel oil (HPKO) at a ratio of 4:6 (*w*/*w*). The melting points, solid fat index (SFI), and melting curves of the alternative products were similar to those of commercial whipping cream fat. The alternative fat had a content of total unsaturated fatty acids 20% higher than that of HPKO. The atherogenic index (AI) of alternative fat was 3.61, much lower than those of whipping cream fat (14.59) and HPKO (1220.3), because of its low atherogenic fatty acid content and high total unsaturated fatty acids. The polymorphic crystal form determined by X-ray diffraction spectroscopy showed that the β’-crystal form was predominant. Therefore, the alternative fat is comparable with whipping cream that requires creaming quality, and has a reduced saturated fat content.

## 1. Introduction

Whipping cream refers to a bubble-containing emulsion. Generally, if the whipping cream contains more than 30% fat, it shows 100–300% of overrun. In the case of whipping cream made from plant oils, palm oil and palm kernel oil are mainly used. With these ingredients, whipping cream normally exhibits better foam stability than with dairy cream [1,2]. Palm kernel oil is a fat that can be obtained from the seeds of palm, and is widely used in confectionery owing to its unique properties as a fat. Further, palm kernel oil contains 80% saturated fatty acids, such as lauric acid (C12:0). Hydrogenated palm kernel oil (HPKO) created via hydrogenation is also used [3]. In hydrogenated oil, *trans* fatty acid and saturated fatty acid contents are increased; these fatty acids are known to increase LDL-cholesterol levels, thereby increasing the risk of cardiovascular diseases [4]. In particular, lauric acid (C12:0), which is present in palm kernel oil and coconut oil in large quantities, is categorized as a saturated fatty acid that increases cholesterol levels [5]. Palm-based vegetable fats mainly contain palmitic (C16:0) and lauric acids, which are considered atherogenic fatty acids [6,7,8]. Therefore, it is desirable to reduce the content of saturated fatty acids in palm-based fat and oil products such as non-dairy creams.

Shea butter, extracted from the fruits of shea trees (*Vitellaria paradoxa* C. F. Gaertner), is used as a fat substitute for cocoa butter and to adjust the physical properties of chocolate. The major fatty acids that compose the triacylglycerol (TAG) portion of shea butter are stearic acid (C18:0) and oleic acid (C18:1). These fatty acids account for approximately 80% of the total fatty acids in shea butter. The remaining fatty acids contained in shea butter are palmitic acid (C16:0) and linoleic acid (C18:2), which are present in low quantities [9]. Although stearic acid is a saturated fatty acid, the rate of its lipolysis by pancreatic lipase may be lower than that of atherogenic saturated fatty acids [10]. Until now, few studies have been conducted on the application of shea butter in whipping cream fat. Therefore, the development of stearic-based whipping cream fat using shea butter was performed in this study.

The main triacylglycerols of shea butter are symmetric 1,3-distearoly-2-oleoyl-*sn*-glycerol (SOS) and asymmetric 1-stearoyl-2,3-dioleoyl-*sn*-glycerol (SOO) [9]. Fats and oils are TAG compounds with different structures, and their crystalline structures change depending on the alignment of each molecule. This phenomenon is called polymorphism, and the representative forms are α-, β’-, and β-crystal forms. Generally, as the form α → form β’ → form β progression occurs, the melting point and stability increase, making the TAG structure compact [11]. Crystal form β mainly appears in the symmetric TAG, and is known to be a desirable crystal form during chocolate preparation, while the β’-crystal form, with small needle-like crystals, appears mainly in the asymmetric TAG [11]. Because of its excellent spreading and creaming quality, it is desirable for use in creams or margarines [6,12]. In lipid modification, acyl migration is a phenomenon wherein the acyl group that is esterified on the TAG molecule moves from position *sn*-1,3 to position *sn*-2 and from position *sn*-2 to position *sn*-1,3 [7]. Such reaction properties can be used to convert symmetric TAGs to asymmetric TAGs for the production of target lipids with desirable physical properties.

The present study aimed to prepare an alternative fat from shea butter with lower saturated fatty acids but with the physical properties of whipping cream. First, the enriched-SOS solid fat, which is a symmetric TAG, was obtained by solvent fractionation using shea butter. Subsequently, SSO, which is an asymmetric TAG displaying mainly the β’-crystal form, was derived through enzymatic acyl migration to enhance the creaming quality. Subsequently, the degree of acyl migration according to reaction time was investigated using the Lipozyme TL IM-catalyzed reaction. The shea butter solid fat obtained by scale-up acyl migration, namely the refined acyl migration reactant (RAMR), was prepared by removing the TAGs with high melting points, such as saturated-saturated-saturated (SSS), and the free fatty acids produced as byproducts. The final product, an alternative whipping cream fat (alternative fat product), was prepared by blending the refined acyl migration reactant (RAMR) and hydrogenated palm kernel oil (HPKO).

The TAG molecular species, fatty acid composition, melting points, and solid fat index of the alternative fat product were analyzed and compared to those of HPKO and whipping cream fat extracted from whipping cream. Crystal forms of the alternative fat product and HPKO were compared using X-ray diffraction spectroscopy (XRD). Whipping cream was then prepared with the alternative fat product to examine its potential for practical use. An overrun test for whipping cream prepared with the alternative fat product was also performed.

## 2. Results and Discussion

### 2.1. Preparation of Shea Butter Solids through Acetone Fractionation and Their Acyl Migration Reaction

#### 2.1.1. Triacylglycerol Regioisomer Separation Analyzed by Ag-High-Performance Liquid Chromatography (HPLC)

**Figure 1:**
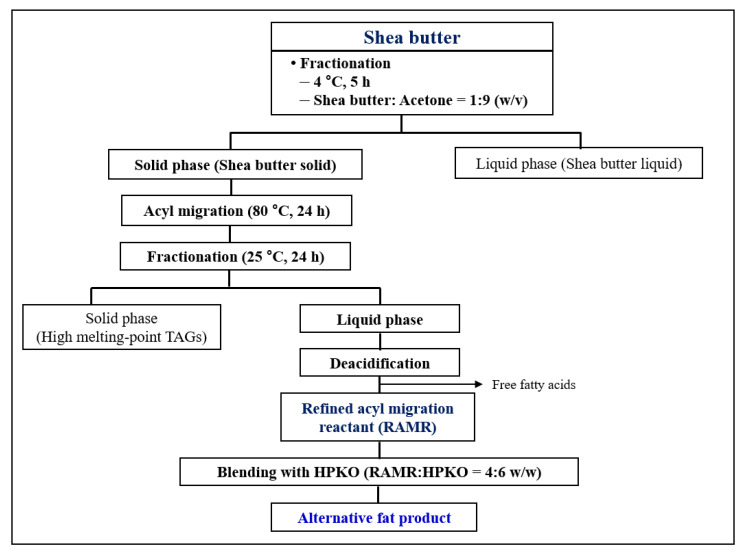
Preparation scheme of the alternative fat for whipping cream.

The objective of this study was to use shea butter to prepare an alternative fat with the physical properties of whipping cream while maintaining contents of saturated fatty acid lower than those of conventional whipping cream. For this purpose, fractionation, enzymatic acyl migration, and blending techniques were applied to prepare an alternative fat for whipping cream from shea butter (Figure 1).

The positional TAG isomer separation results of shea butter, shea butter liquid and solid fractions, and acyl-migrated shea butter solids (0, 3, 24, and 100 h) analyzed using a normal-phase HPLC-evaporative light-scattering detector (ELSD) with an Ag column are summarized in Table 1. Silver-ion HPLC provides regioisomer separation for TAG molecules according to the number, configuration, and position of double bonds in fatty acids [13]. Using this method, the degree of acyl migration of shea butter solid used as a substrate was compared according to the enzymatic reaction time (0, 3, 24, and 100 h). The shea butter consisted of 67.8 area% saturated-unsaturated-saturated (SUS) TAGs and 31.3 area% saturated-unsaturated-unsaturated (SUU) TAGs (Table 1), similar to the results of a previous study [14]. Shea butter solid obtained after acetone-fractionation at 4 °C showed that symmetric TAG content of SUS was 92.5 area% and asymmetric TAG content of SUU was drastically reduced to 6.9 area% from 31.3%. In the shea butter liquid fraction (Table 1), symmetric TAGs such as SUS were present only at 3.6 area%, and most of the asymmetric TAGs presented as SUU type (96.4 area%).

**Figure 2:**
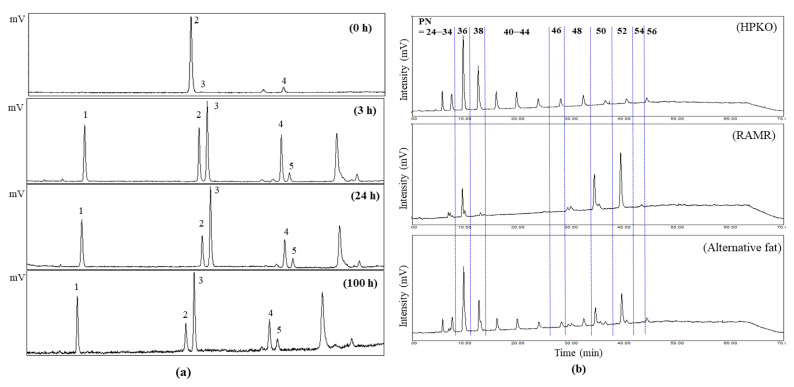
Triacylglycerol (TAG) regioisomer chromatograms of acyl migration reactants (0, 3, 24, and 100 h) obtained via Ag-HPLC analysis (**a**) and TAG molecular species chromatograms of hydrogenated palm kernel oil (HPKO), refined amyl-migration reactant (RAMR), and alternative fat product (RAMR:HPKO = 4:6 blend, *w*/*w*) obtained from analysis of the revered-phase HPLC (**b**). TAG regioisomer chromatograms: 1. SSS (saturated-saturated-saturated); 2. SUS (saturated-unsaturated-saturated); 3. SSU (saturated-saturated-unsaturated); 4. SUU (saturated-unsaturated-unsaturated); 5. USU (unsaturated-saturated-unsaturated).

The stability of whipping cream is extensively affected by the solid fat content or solid fat index, molecular structure, and crystal form of fat. In order to create a creamy structure in fat, the β’-crystal form is needed, which is derived from asymmetric TAGs such as saturated-saturated-unsaturated (SSU). Therefore, in order to induce acyl migration for stearic-oleic-stearic triacylglycerol (SOS), which is abundant in the shea butter solid, into stearic-stearic-oleic (SSO), it was reacted for 3, 24, and 100 h with Lipozyme TL IM, of which the water activity (*aw*) increased to 0.73. Their acyl migration degrees are presented in Table 1 and Figure 2a. Since moisture is associated with enzyme activity and asymmetric TAG formation, it is an essential factor in enzyme reactions. The higher the water activity in the enzyme and the longer the reaction with water activity, the higher the acyl migration reaction rate [7,15]. In the result of acyl migration of shea butter solid obtained from acetone fractionation, the saturated-saturated-saturated (SSS) TAG, which was not detected before acyl migration, increased to 23.1 area% after 3 h of reaction, followed by no significant increase after the reaction time was extended (*p* > 0.05). In the case of symmetric SUS, it was 92.5 area% before acyl migration but decreased to 20.1 area% after 3 h of reaction. During the acyl migration reaction for 24 and 100 h, there was a significant decrease of 12.1–13.3 area% (*p* < 0.05). The asymmetric TAG SSU, which showed a low value of 0.6 area% before the reaction (0 h, shea butter solid fraction), increased as the reaction time was extended, reaching 31.2 area% (3 h), 41.6 area% (24 h), and 38.5 area% (100 h). SUU, another asymmetric TAG, was 6.9 area% before the reaction, increasing to 21.5 area% after 3 h of reaction and decreasing to 16.3 and 15.7 area% after 24 and 100 h of reaction, respectively (Table 1). As the acyl migration reaction time increased, the production of SSS TAG as a side reaction also increased.

Generally, acyl migration takes place more as the reaction time increases [7,15]. However, according to our results, the highest acyl migration was observed after 24 h of reaction. In addition, relatively low contents of SSS and SUS TAGs were observed. Thus, the scaled-up reaction of acyl migration using a shea butter solid was carried out for 24 h with Lipozyme TL IM of *aw* 0.73. The scale-up reactant (acyl-migrated shea butter solid, 24 h) was purified through deacidification for removal of byproducts and fractionation for removal of high-melting-point TAG to obtain the refined acyl migration reactant (RAMR). The prepared RAMR comprised 19.5 area% SUS, 47.9 area% SSU, 24.5 area% SUU, and 8.1 area% USU. It was then used for further experiments.

#### 2.1.2. Triacylglycerol Molecular Species Analyzed Using Reversed-Phase HPLC

In TAG species analysis using reversed-phase HPLC equipped with an evaporative light-scattering detector (ELSD), the TAG molecular composition of fats and oils can be separated based on both the double bond number (ND) and carbon chain length (total carbon number; CN) in fatty acid molecules. TAG elution depends on partition number (PN), which is calculated using CN and ND [7]. In this RP-HPLC system, regioisomeric TAGs such as SOS (1,3-stearol-2-oleolylglycerol; stearic-oleic-stearic TAG) and SSO (1,2-stearol-3-oleolylglycerol; stearic-stearic-oleic TAG) cannot be separated. TAG regioisomer separation was carried out using Ag-HPLC, as described above. Therefore, two HPLC systems were used to separate TAG composition. The regioisomeric structure of TAG in fats plays an important role in fats’ physical properties such as fat crystals, melting points, and textures in lipid-based processed products [12,16]. The TAG molecular species of the HPKO, shea butter, shea butter solid and liquid fractions, RAMR, and alternative fat products were analyzed using RP-HPLC, and their proposed TAG compositions are summarized in Table 2. The partition numbers (PNs) for the peaks of each TAG molecule are shown in Figure 2b and Table 2. Generally, shea butter is composed mainly of SOS and SOO (stearic-oleic-oleic TAG) as major TAGs, and palmitic-oleic-oleic TAG (POO) and palmitic-oleic-stearic TAG (POS) [17]. The PN of the TAG composition in the shea butter used in this study was distributed at around PN = 48–54; in particular, TAG with PN = 52 was 55.5 area%, while TAG with PN = 50 was 32.0 area%.

According to the results of Ag-HPLC (Table 1) and a study by Zhang et al. [18], TAG at PN = 52 mostly contained SOS, while TAG at PN = 50 contained SOO. In the shea butter solid, TAG with PN = 52 was 92.7 area%, which was a higher value than that of shea butter, and most of them were SOS (Table 2). In addition, the total area% of SOO and POS with PN = 50 was 6.6%, which was distinctly lower than that in the shea butter. In contrast, the shea butter liquid had a very high amount (82.8 area%) of SOO at TAG with PN = 50. This is because TAGs with relatively high melting points, such as SOS, were present in the shea butter solid after acetone fractionation at 4 °C, while TAGs with a low melting point, such as SOO, were present in the shea butter liquid [18]. In the case of the RAMR, the contents of SSO and SOS with PN = 52 were 51.1%, while those of SOO and OSO were 30.8 area%. Because enzymatic acyl migration occurred in the shea butter solid, including the high SOS content of 92.7 area%, not only SSO TAGs but also trisaturated TAGs with high melting points were produced, as shown in Table 1. Therefore, fractionation and deacidification using a 24 h acyl migration reactant of shea butter solid were performed to remove high-melting-point TAGs and free fatty acids in order to obtain the RAMR.

SOS TAG existed mainly in the shea butter solid; this is known as β-tending TAG, and results in a stable β-crystal form [7,12]. In contrast, the SSO TAG, which presents as β’-tending TAG in the RAMR, provides a desirable β’-crystal stable form for the creaming properties of fat.

The TAG species of HPKO, which was used for blending to prepare the alternative fat product, were distributed mainly in a range of PN = 24–44. This is because large amounts of medium-chain fatty acids were present in HPKO [7]. In particular, TAG which was assumed to be LaLaLa (lauric-lauric-lauric TAG), with PN = 36, was found to be as high as 26.2 area% (Table 2). The alternative fat was prepared by blending RAMR and HPKO (4:6, *w*/*w*) to replace the physical properties of whipping cream fat. The PNs of TAG species in the alternative fat product ranged from to 24–56, indicating a variety of TAG species.

### 2.2. Fatty Acid Composition and Melting Point

The desirable smooth texture and spreadability of whipping cream can be influenced by the fat crystal form (especially β’-crystal): a type of polymorphism [19]. To obtain the desirable β’-crystal form, the fatty acid composition of fats to be applied for whipping cream is an important factor [19]. The complex composition of fatty acids and the asymmetric structure of TAG tend to result in a typical β’-crystal form [7,19]. In the fatty acid composition of the extracted fat from commercial whipping cream, the composition of various fatty acids (C4:0–C18:3*n*3, mainly C12:0 34.44%) was determined to be 91.62% of total saturated fatty acids and 7.59% of total unsaturated fatty acids (Table 3). The fatty acid composition of the HPKO used as a blending fat for the production of the proposed alternative fat was mainly composed of C12:0 (lauric acid, 46.50%), C18:0 (stearic acid, 19.99%), C14:0 (myristic acid, 16.69%), and C16:0 (palmitic acid, 8.77%), resulting in 99.73% total saturated fatty acids and 0.1% total unsaturated fatty acids (Table 3). The fatty acid composition of whipping cream fat was similar to that of HPKO. The content of total unsaturated fatty acids detected in whipping cream fat might have been due to additional fat rather than HPKO during the processing of the whipping cream. The atherogenicity index (AI), based on the content of lauric acid (C12:0), myristic acid (C14:0), and palmitic acid (C16:0) in fats, is related to coronary heart disease [7,8]. As dietary intake of these three atherogenic fatty acids increases, negative health effects occur. The high content of total unsaturated fatty acids can be an important factor in reducing the atherogenicity index. The AI values of whipping cream fat and HPKO were 14.59 and 1220.3, respectively (Table 3). This is related to the fact that the total unsaturated fatty acid content (0.10%) of HPKO is much lower than that of whipping cream fat (7.59%).

The major fatty acids of the shea butter solid obtained through acetone fractionation from shea butter were stearic acid (55.80%) and oleic acid (34.37%). The total saturated fatty acid and total unsaturated fatty acid contents were 61.09% and 38.75%, respectively. The AI of the shea butter solid was very low (0.09) because it did not contain atherogenic fatty acids and had a relatively high content of total unsaturated fatty acids.

In the fatty acid composition of the RAMR, stearic acid (44.28%) and oleic acid (45.04%) were found to be the major fatty acids. Therefore, the total unsaturated fatty acid content (50.71%) in the RAMR was higher than that of the shea butter solid(38.75%) before acyl migration, because saturated-saturated-saturated TAG (SSS), with a high melting point and composed of saturated fatty acids, was removed during purification, as shown in Table 1 and Table 2. The AI (0.07) of the RAMR was also low, similar to that of the shea butter solid.

The slip melting (SM) and complete melting (CM) points of whipping cream fat, HPKO, shea butter solid, and RAMR are presented in Table 3. In the case of the shea butter solid, the slip melting point and complete melting point were measured as 34.3 °C and 38.5 °C, respectively. The complete melting point of the RAMR was 41.5 °C. This might be due to increases in the contents of asymmetric TAG and high melting-point TAGs (PN = 50 and 54), including saturated fatty acids after acyl migration (Table 2). In the case of HPKO, the slip melting point was 39.3 °C, while the complete melting point was 40.3 °C. In the fat extracted from the whipping cream, the slip melting point was measured as 37.5 °C, while the complete melting point was measured as 39 °C.

### 2.3. Melting and Crystallization Curves and Solid Fat Index (SFI)

**Figure 3:**
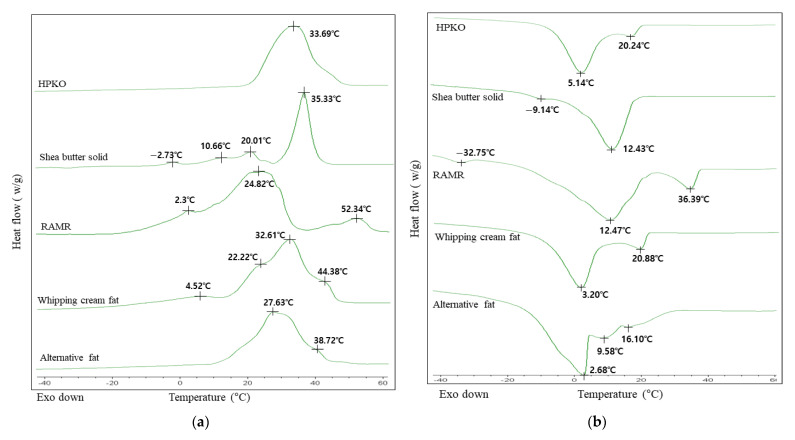

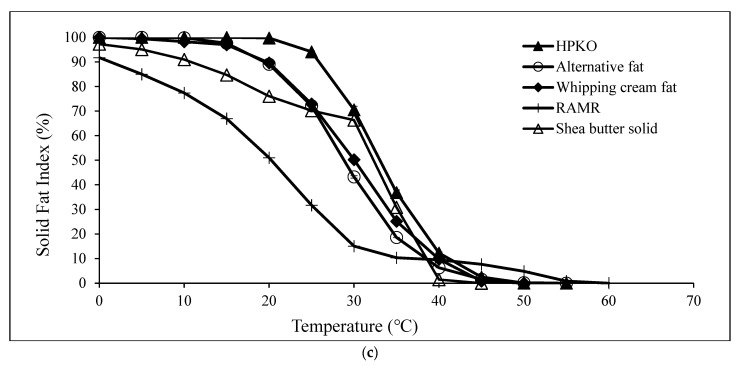
DSC melting (**a**) and crystallization (**b**) curves and solid fat index (SFI, (**c**)) of hydrogenated palm kernel oil (HPKO), refined amyl migration reactant (RAMR), whipping cream fat, and alternative fat (HPKO:RAMR = 6:4 blend, *w*/*w*).

The melting and crystallization behavior of fats used as whipping cream is also an important factor in providing desirable physical properties such as plasticity, texture, and mouthfeel. In general, these physical properties can be measured using differential scanning calorimetry (DSC). The melting (endothermic) and crystallization (exothermic) curves of the shea butter solid, RAMR, HPKO, whipping cream fat, and alternative fat are presented in Figure 3a,b. The melting curve (Figure 3a) of HPKO, which was used for blending in preparation of the alternative fat product, showed a sharp endothermic peak at 33.69 °C. Its crystallization curve had one maximum exothermic peak at 5.14 °C and a small exothermic peak at 20.24 °C (Figure 3b). The melting curve of the shea butter solid (Figure 3a) had the largest endothermic peak at 35.33 °C, which was shifted to a higher temperature compared with that of the shea butter. This might be because the shea butter solid contained more TAG, which has a relatively high melting point, after fractionation. On the melting curve of the RAMR (Figure 3a), two major endothermic peaks were observed at 24.82 °C, along with another small peak at 52.34 °C. The crystallization curve (Figure 3b) of the RAMR showed two major exothermic peaks at 12.47 °C and 36.39 °C, with its melting point shifted to a higher temperature than that of the shea butter solid. Generally, asymmetric TAGs such as SSO and PPO have a higher melting point than symmetric SOS and POP [7]. Therefore, symmetric TAG, which occupied most of the shea butter solid, was converted to asymmetric TAG after acyl migration, explaining the fact that the complete melting point of the RAMR, which had a lower total saturated fatty acid content than the shea butter solid, increased (Table 3). On the melting and crystallization curves of whipping cream fat (Figure 3a,b), a main endothermic peak appeared at 32.61 °C and two major exothermic peaks appeared at 3.20 °C and 20.88 °C. Patterns of exothermic and endothermic curves between the HPKO and the commercial whipping cream fat were not matched, which might have been because the whipping cream fat extracted from commercial whipping cream contained HPKO as well as other processed fats. The endothermic and exothermic peak profiles of the whipping cream fat were generally similar to those of HPKO (Figure 3a,b).

The solid fat index (SFI) affects physical properties of fats and oils, such as mouthfeel and spreading, and is thus an important parameter for evaluating the physical properties of fats and oils in the fatty food industry [7,20]. The SFI measured by DSC is the solid–liquid ratio in a fat. It was determined by partial integration at various temperatures based on the melting curve. The SFI values of the shea butter solid, RAMR, HPKO, whipping cream fat, and alternative fat are shown in Figure 3c. In the case of the shea butter solid obtained through fractionation, the SFI value (%) gradually decreased from 97.25% to 70.20% up to 30 °C, and then abruptly decreased until 40 °C, reaching 0% at 45 °C. The SFI profile of the shea butter solid composed of 92.7% SOS TAG showed lower values than those of HPKO and whipping cream fat in the temperature range of 0–20 °C.

For the RAMR, as the temperature increased, the SFI values gradually decreased, but at a relatively higher temperature, it reached 0%. In contrast, HPKO showed a higher SFI value compared to other fats at temperatures in the range of 0–40 °C. In particular, at 30–40 °C, the SFI value abruptly decreased. At temperatures higher than 55 °C, it was 0%. For the fat extracted from whipping cream, the SFI value was high in the range of 0–40 °C, compared with that of the RAMR. Therefore, if a certain amount of HPKO with relatively high solid fat were blended with the RAMR, it was expected to have a similar SFI value to the whipping cream fat.

### 2.4. Preparation of Alternative Fat by Blending of Refined Acyl Migration Reactant (RAMR) and Hydrogenated Palm Kernel Oil (HPKO)

The objective of this experiment was to prepare an alternative fat product using shea butter which had the physical properties of whipping cream but with lower saturated fat content. Whipping cream was originally formulated from anhydrous milk fats. Palm-based vegetable fats, such as palm oil, palm kernel oil, and their hydrogenated fats are used because of their high cost and low foam stability [20]. The main fatty acids that exist in palm-based fats, such as palmitic (C16:0) and lauric (C12:0) acids, are considered atherogenic fatty acids [6,7,8]. Stearic acid (C18:0), a major saturated fatty acid in shea butter solid, has been shown to have some resistance (lower lipolysis) to pancreatic lipase [10]. Therefore, in this study, stearic-based whipping cream fat was developed as an alternative to palm-based fats.

In this study, the RAMR was blended with HPKO, which is a major ingredient of the whipping cream, enabling the RAMR to display physical properties similar to whipping cream fat. The melting and crystallization curves of the alternative fat obtained by blending RAMR and HPKO at a ratio of 4:6 (*w*/*w*) are presented in Figure 3a,b. In the melting curve of the alternative fat (Figure 3a), the main endothermic peak at 27.63 °C showed a narrower peak than the RAMR, and the small endothermic peak at 38.72 °C decreased relatively more than that of the RAMR at 52.34 °C. In the crystallization curve of alternative fat (Figure 3b), exothermic peaks from 2.68 °C to 16.10 °C appeared, similarly to the exothermic peaks (3.20 and 20.88 °C) of whipping cream fat. From the crystallization curve, we predicted that the alternative fat would have a higher slip melting point than RAMR because of its lower exothermic peaks (Table 3 and Figure 3b).

In the SFI value presented in Figure 3c, the SFI value of the alternative fat blended at 4:6 (*w*/*w*) was not significantly different from that of whipping cream fat until the temperature reached 25 °C. The SFI values were slightly lower than those of whipping cream fat at 25–40 °C, but were generally similar in shape. By blending HPKO with a relatively high SFI with the RAMR, an SFI similar to that of whipping cream fat was obtained. When the RAMR and HPKO were blended at 4:6 (*w*/*w*), the physical properties of alternative fat were very similar to those of the whipping cream fat, according to the melting and crystallization curves and the SFI. Therefore, the physicochemical characteristics of the alternative fat, such as fatty acid composition, TAG molecular species, and fat crystal form, were measured. In addition, whipping creams were prepared using an alternative fat product and HPKO, respectively. Their foam stability was compared and validated through an overrun test.

### 2.5. Physicochemical Characteristics of Alternative Fat

TAG molecular species of the alternative fat product were distributed broadly with PN = 24–56, in which TAG with PN = 24–44 was derived from HPKO, while TAG with PN = 50–52 was derived from the RAMR (Table 2). The slip melting point of the alternative fat, which had a similar SFI pattern as the whipping cream fat, was 37.5 °C, while the complete melting point was 39.3 °C, which was similar to that of the whipping cream fat. In the alternative fat, saturated fatty acids accounted for 79.33%, including lauric (27.78%), myristic (9.94%), palmitic (6.57%), and stearic acids (29.82%), while unsaturated fatty acids such as oleic acid (18.19%) accounted for 20.51% (Table 3). Therefore, in the alternative fat, the content of unsaturated fatty acids was approximately 13% higher than that of whipping cream fat. Furthermore, the AI of alternative fat was much lower (3.61) than that of either whipping cream fat (14.59) or HPKO (1220.3) because of its relatively low content of atherogenic fatty acids and its relatively high content of total unsaturated fatty acids (Table 3).

In general, three types of polymorphic form (α-, β’-, and β-crystal forms) are representative crystalline structures of fats and oils. The crystal form α is the most unstable and shows single spacing at 4.15 Å when analyzed by X-ray diffraction spectroscopy (XRD), while the β’-crystal form is mainly found in foods like margarines and shortenings and shows short spacing at 3.8 Å and 4.2 Å [21]. The β’-crystal form tends to coagulate, making a net structure that contains air, and thus can provide creamy characteristics [19]. Meanwhile, the β-crystal form is found in foods such as chocolate, is the most stable, and exhibits strong short spacing at 4.6 Å [6,19,20]. Since the crystal form of fat in the foods like whipping cream affects the capture and stabilization of air cells in cream, the β’-crystal form is desirable [22]. The XRD structural analysis results for these crystal forms are presented in Table 4. HPKO showed short spacings at 4.2 Å and 3.8 Å, but not at 4.6 Å, indicating that HPKO had the β’-crystal form. In the alternative fat, crystalline structure was presented at 4.6 Å, 4.2 Å, and 3.8 Å with relatively strong intensities at 4.2 Å and 3.8 Å, which means the β’-crystal form was dominant over the β-crystal form. Therefore, it can be said that in the case of the alternative fat, the β’-crystal form was more prevalent.

**Figure 4:**
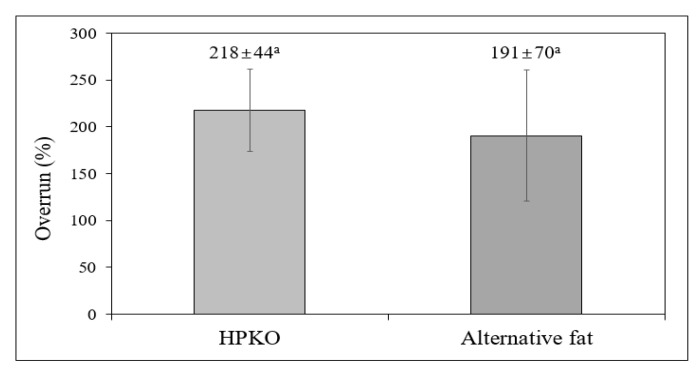
Overrun (%) of whipping creams prepared with hydrogenated palm kernel oil (HPKO) and alternative fat (HPKO:RAMR = 6:4 blend, *w*/*w*).

Figure 4 shows the overrun test (%) of the whipping creams prepared from the alternative fat and HPKO. The overrun represents the degree of air captured inside a whipping cream. It is generally known that the stability and hardness of whipping cream reach a maximum level at maximum overrun [1]. The overruns of the whipping creams made of HPKO and of alternative fat were 217.5% and 190.5%, respectively, with slightly higher values in the whipping cream made from HPKO, but the difference was not significant (*p* > 0.05). Therefore, we expect that the alternative fat developed in this study could be used in the manufacture of whipping creams with reduced saturated fat content.

## 3. Materials and Methods

### 3.1. Materials

Hydrogenated palm kernel oil (HPKO) was provided by Lotte (Seoul, Korea), while shea butter and whipping cream were purchased from the local market. Lipozyme TL IM was purchased from Novozymes (Seoul, Korea). Fatty acid methyl ester (FAME) standard was obtained from Supelco (Bellefonte, PA, USA) as 37 component FAME Mix. HPLC-grade reagents were used for all instrumental analyses. Lactylated monoglyceride, sodium caseinate, xanthan gum, sodium stearoyl lactylate, and monoglyceride for the preparation of whipping cream emulsion were provided by local companies, while methyl cellulose was purchased from Sigma-Aldrich Chemical Co. (St Louis, MO, USA).

### 3.2. Extraction of Whipping Cream Fat from Commercial Whipping Cream

Crude whipping cream fat was extracted from commercial whipping cream using an acid hydrolysis method [23,24,25]. First, approximately 1 g of whipping cream was placed in a 50 mL vial, and 2 mL of pyrogallol ethanol solution (50 mg/mL) was added to the vial with the sample as an antioxidant. Ten milliliters of 8.3 M HCl solution were added for acid hydrolysis; the mixture was vortexed for 30 s and then reacted for 1 h in a shaking water bath set at 80 °C and 200 rpm. Each vial was vortexed every 20 min to promote sufficient reaction. After the reaction, the reactants were cooled with cold water. For crude fat extraction, 15 mL of diethyl ether was added and the mixture was stirred for 1 min and then centrifuged for 3 min at 2500 rpm using a centrifuge (HA-1000-3, Hanil Science Industrial Co., Ltd., Incheon, Korea). The supernatant obtained by centrifugation was purified by passing through an anhydrous sodium sulfate column to remove residual moisture, and it was collected in a new 50 mL vial. The same procedure was repeated after the addition of 15 mL of petroleum ether. The supernatant collected in the 50 mL vial was removed with nitrogen gas, and crude fat was obtained.

### 3.3. Pre-Equilibration for Water Activity (a_w_) of Lipozyme TL IM

A saturated salt solution for pre-equilibration of the water activity of Lipozyme TL IM was prepared using (NH_4_)_2_SO_4_ (a_w_ = 0.80) [26]. To equilibrate the water activity, Lipozyme TL IM was left inside a sealed container in an incubator (SI-900R, Jeio Tech, Daejeon, Korea) at 25 °C for 24 h. Water activity of the enzyme was measured using a water activity tester (AQUA LAB series 3TE, Decagon Devices Inc., USA) at 25 °C. The water activity of Lipozyme TL IM was 0.73.

### 3.4. Acetone Fractionation of Shea Butter for Preparation of Shea Butter Solids

Shea butter stearin (solid fat of shea butter) was obtained by solvent fractionation using acetone, as shown in Figure 1. For fractionation, 50 g of shea butter was placed in a 500 mL beaker and completely melted at approximately 60 °C by adding it to acetone at a ratio of 1:9 (*w/v*). The uniformly melted sample solution was then left to crystalize for 5 h in an incubator (SI-900R, Jeio Tech, Dajeon, Korea) at 4 °C. From the mixture, solid (shea butter solid fraction) and liquid (shea butter liquid fraction) phases were separated using a vacuum filtration device. The acetone from the separated solid phase was entirely removed using a rotary vacuum evaporator and nitrogen flush [7]. The shea butter solid fat obtained in this way was further used for the enzymatic acyl migration reaction.

### 3.5. Enzymatic Acyl Migration of Shea Butter Solids and Production of Refined Acyl Migration Reactant (RAMR)

Acyl migration of the shea butter solids (10 g) was carried out in a 100 mL Erlenmeyer flask using 20 wt% (*w*/*w* total reactants) of Lipozyme TL IM pre-equilibrated at *a_w_* = 0.73. The reaction was performed in a 185 rpm shaking water bath (BS-21, Lab Companion, Ramsey, MN, USA) set at 80 °C, and the reaction time was set to 3, 24, and 100 h to optimize the degree of acyl migration. After reaction at each reaction time, TL IM enzymes were separated from the reactant using a 0.5 μm PTFE syringe membrane filter. The filtrate of the acyl-migrated reactants was isolated and obtained. For the scaled-up production of acyl-migrated reactants, the reaction was conducted with 25 g of shea butter solids and 20 wt% of Lipozyme TL IM for 24 h under the same reaction conditions. Acetone fractionation and deacidification of the reactants were carried out to remove the TAGs with high melting points (such as saturated-saturated-saturated) and the free fatty acids produced as byproducts from the reactants. Finally, the refined acyl migration reactant (RAMR) was obtained as follows. For fractionation, the acyl migrated reactant was entirely melted in acetone equivalent to 15 times the weight (*v*/*w*) of reactants and was stirred sufficiently. The reactant solution was then left for 24 h in an incubator in which the temperature was maintained at 25 °C with no agitation. After fractionation, the liquid and solid phases were separated using a vacuum filtration device. The liquid fraction was obtained after separation from the precipitated solid fraction with high melting points. The liquid fraction was completely dried using a rotary vacuum evaporator and nitrogen flush. For the removal of free fatty acids generated as byproducts, the dried liquid fraction was deacidified by alkali neutralization process [27]. Deacidification of the liquid fraction was carried out in a separating funnel with enough *n*-hexane to dissolve the fat. A few drops of 0.1% phenolphthalein ethanol solution were added to the separating funnel as an indicator. Finally, titration using a 0.5 N KOH ethanol solution was performed until a pink color appeared. After titration, *n*-hexane was added to completely separate the layers. The obtained *n*-hexane layer was washed several times with deionized water at approximately 40 °C. The washed *n*-hexane layer was then passed through an anhydrous sodium sulfate column to remove residual moisture and impurities. The *n*-hexane in the liquid fraction was evaporated by a rotary vacuum evaporator and a nitrogen flush to obtain the refined acyl migration reactant (RAMR) [27]. The RAMR was stored at −20 °C for further analysis.

### 3.6. Analysis of Fatty Acid Composition, Triacylglycerol Molecular Species, and Triacylglycerol Regioisomer Separation

For analysis of fatty acid composition, samples were methylated into fatty acid methyl esters (FAMEs) using potassium hydroxide in a methanol solution (0.5 N KOH in anhydrous methanol) according to previous studies with some modifications [7,23]. Methylation was carried out for 10 min in an orbital shaking water bath set at 85 °C and 200 rpm. Analysis of FAMEs for fatty acid composition was performed using gas chromatography (GC, Agilent 6890 series, Santa Clara, CA, USA) with a flame ionization detector and an SP^TM^-2560 fused silica capillary column (100 m × 0.25 mm, i.d. 0.2 μm film thickness, Supelco, Bellefonte, PA, USA) [23,25]. The carrier gas was high-purity He at a flow rate of 0.7 mL/min.

For the analysis of TAG species composition, reversed-phase HPLC was used, based on a previous study [7]. A Nova-Pak C18 column (60 Å, 4 μm, 3.9 × 50 mm i.d., Waters, Milford, Ireland) and SEDEX 75 evaporative light-scattering detector (ELSD, Dedere Alfortvill, France) were used for the separation of TAG species. The partition number (PN) of each TAG composition was calculated using the following equation [7]: PN = total carbon number (CN) − 2 × total number of double bonds (ND).

Composition of symmetric and asymmetric TAG isomers was analyzed using Ag-HPLC equipped with a silver ion column (Chrom Spher 5 lipids 250 × 4.6 mm i.d., Varian, Netherlands) and a SEDEX 75 evaporative light-scattering detector (ELSD, Dedere Alfortvill. France) set at 40 °C and a pressure of 2.2 bar. Solvent A (*n*-hexane:iso-propanol:acetonitrile = 100:0.1:0.1, by volume) and solvent B (*n*-hexane:iso-propanol:acetonitrile = 100:1:1, by volume) were eluted with a binary gradient at a flow rate of 1 mL/min with the following linear gradient profile: 100% A for 5 min, reduced to 80% A over 45 min, 50% A over 10 min, 50% A for 1 min, 100% A for 2 min, and 100% A for 8 min [28].

### 3.7. Melting Points, Solid Fat Index (SFI), and Melting and Crystallization Curves

The melting points of fats were measured via the capillary tube method according to the AOCS Official Method Cc1-25 [29]. The solid fat index (SFI) and melting and crystallization profiles of fat samples were determined by differential scanning calorimetry (DSC 2010, TA Instruments, New Castle, DE, USA) according to a previous study [7]. The SFI value was determined from the melting point obtained through DSC analysis by using changes in unit energy at each temperature zone (5 °C) against total ∆H (J/g, enthalpy) within the total melting section.

### 3.8. Preparation of Alternative Fat Product

The reactant obtained by acyl migration for 24 h was fractionized and deacidified, and then obtained as a refined acyl migration reactant (RAMR). The RAMR was blended inside a double jacket with HPKO at a ratio of 4:6 (wt%) at 55 °C. An alternative fat blend with a ratio of 4:6 wt% was set as the final product for further experiments (Figure 1). To investigate the physicochemical properties of the alternative fat product, fatty acid composition, TAG molecular species, melting points, DSC analysis, and overrun were analyzed using the methods described above.

### 3.9. Polymorphism by X-ray Diffraction Spectroscopy

Polymorphic forms (β’ and β) of HPKO and the alternative fat product were measured with multipurpose X-ray diffraction with D/Max-2200 Model Ultima/PC (Ultima III, Rigaku Int. Corp. Tokyo, Japan), at room temperature [6,22]. The X-ray tube was Cu–Ka at a voltage of 40 kV and current of 40 mA. Data were obtained in the 2θ range of 18–32°. The X-ray wavelength (*λ*) used to measure the X-ray diffraction pattern was 1.5406 nm. Each sample was tempered in a rectangular plastic mold at 4 °C for 24 h for analysis.

### 3.10. Whipping Cream Preparation and Overrun Test

First, emulsions were prepared using the alternative fat product and HPKO as follows: The overrun (%) of whipping creams prepared with each emulsion was analyzed and compared between the alternative fat product and HPKO.

The ingredients and their blending ratios (wt%) of the O/W emulsions prepared for whipping cream are listed in Table 5. First, the aqueous phase was prepared by adding corn syrup, sodium caseinate, methyl cellulose, and sodium stearoyl lactylate (SSL) sequentially into distilled water at 80 °C, and was dissolved by heating on a stirrer. Then, in order to dissolve xanthan gum into the aqueous phase, preliminary homogenization was performed using a Silverson homogenizer (L4RT, Silverson Machines Ltd., Chesham, UK) at 8000 rpm for 10 min during heating. The oil phase was prepared by completely dissolving the lipophilic emulsifier monoglyceride (0.5 g) and lactylated monoglyceride (LMS, 0.2 g) into 25 g fat (final alternative fat product or HPKO) during heating. The prepared aqueous phase and preheated oil phase were premixed together by performing preliminary homogenization with a Silverson homogenizer at 8000 rpm for 10 min. The pre-mixed emulsion was immediately homogenized using a high-pressure homogenizer (M-110Y, Microfudics, MA, USA) at 1500 psi. After emulsification, the emulsion was tempered in dry ice for 30 min and then stored in a refrigerator at 4 °C for 24 h. The emulsion was then removed using a mini-whipper at a maximum speed of 5 for 6 min. The overrun of the whipped emulsion was calculated by weighing it before and after whipping in a cup with a standardized volume (100 mL) using the following equation [1]:

$$\%\;\mathrm{Overrun}=\;\frac{\mathrm{Weight}\;\mathrm{of}\;\mathrm{unwhipped}\;\mathrm{emulsion}-\mathrm{Weight}\;\mathrm{of}\;\mathrm{whipped}\;\mathrm{emulsion}}{\mathrm{Weight}\;\mathrm{of}\;\mathrm{whipped}\;\mathrm{emulsion}}\;\times 100$$

### 3.11. Statistical Analysis

The experimental results are expressed as mean ± standard deviation, and Duncan’s multiple range test was performed with Statistical Analysis System 9.2 (SAS Institute Inc., Cary, NC, USA). The significance difference of the means was determined to be *p* < 0.05.

## 4. Conclusions

In this study, shea butter was used to develop an alternative fat with physical properties similar to those of commercially available whipping cream while presenting reduced saturated fat content. A considerable amount of symmetric TAG (SOS 92.7 area%) was present in the shea butter solid obtained by acetone fractionation. Because the β’-crystal form is desirable for foods like whipping cream and the β’-crystal form is derived from asymmetric TAGs such as saturated-saturated-unsaturated (SSU), acyl migration reaction was induced to convert symmetric SOS TAG into asymmetric SSO TAG. The water activity of Lipozyme TL IM, which was used for acyl migration, was raised to 0.73, and the reaction was carried out for 3, 24, and 100 h. After reaction for 24 h, the content of SSU was approximately 3.4-fold higher than that of SUS. Therefore, after the scaled-up production of the acyl migration reaction, high-melting-point TAGs and free fatty acids were removed to prepare the RAMR.

The RAMR and HPKO were blended at a ratio of 4:6 (*w*/*w*) to obtain an alternative fat with a similar SFI to whipping cream fat. The results showed that the SFI pattern was the most similar to whipping cream fat at a blending ratio of 4:6 (*w*/*w*). Similar complete melting points were observed for whipping cream fat and alternative fat at 39.0 °C and 39.3 °C, respectively.

TAG molecular species in the alternative fat were broadly distributed, showing a PN range of 24–56; the TAG with PN = 24–44 was derived from HPKO, while the TAG with PN = 50–52 was derived from the RAMR. The saturated fatty acid content was 79.33% in the alternative fat, including 27.78% lauric acid, 9.94% myristic acid, 6.57% palmitic acid, and 29.82% stearic acid, and unsaturated fatty acids such as oleic acid (18.19%) at approximately 20.51%. Therefore, the alternative fat had an unsaturated fatty acid content approximately 20% greater than that of HPKO. The AI of the alternative fat was 3.61 because of the low content of atherogenic fatty acids and the relatively high content of total unsaturated fatty acids, which was much lower than those of whipping cream fat (14.59) and HPKO (1220.3).

Emulsions of alternative fat and HPKO were prepared. They were whipped to determine the practical feasibility of the alternative fat for the industry. The overrun of the whipping cream prepared from HPKO was on an average 217.5%, while it was on an average 190.5% for the whipping cream made of the alternative fat, but without a significant difference (*p* > 0.05). The crystalline structure determined by XRD showed that the β’-crystal form was predominant. Therefore, the alternative fat proposed in this study would be comparable with whipping cream that requires creaming quality, since the alternative fat had similar SFI and β’-crystal forms while displaying lower saturated fat content than commercially available whipping creams.

## Figures and Tables

**Table 1:** Positional triacylglycerol (TAG) comparison (area%) of fractionations (liquid and solid) of shea butter, acyl migration reactants of shea butter solid prepared from different reaction times (0, 3, 24, and 100 h), and refined acyl migration reactant (RAMR).

-	Acetone Fractionation (4 °C, 5 h)			Lipozyme TL IM (*aw*: 0.73)			RAMR
-	Shea Butter	Shea Butter Liquid Fraction	Shea Butter Solid Fraction (0 h)	3 h	24 h	100 h	
SSS ^(1)^	ND	ND	ND ^(6)^	23.1 ± 0.9 ^a^	25.3 ± 0.9 ^a^	25.4 ± 0.5 ^a^	ND
SUS ^(2)^	67.8 ± 0.4	3.6	92.5 ± 2.1 ^a^	20.1 ± 1.3 ^b^	12.1 ± 2.5 ^c^	13.3 ± 1.2 ^c^	19.5 ± 3.0
SSU ^(3)^	0.8 ± 0.5	ND	0.6 ± 0.2 ^d^	31.2 ± 1.2 ^c^	41.6 ± 0.5 ^a^	38.5 ± 1.0 ^b^	47.9 ± 2.1
SUU ^(4)^	31.3 ± 0.1	96.4	6.9 ± 1.9 ^c^	21.5 ± 2.3 ^a^	16.3 ± 1.8 ^b^	15.7 ± 0.5 ^b^	24.5 ± 0.2
USU ^(5)^	ND	ND	ND	4.1 ± 1.1 ^b^	4.8 ± 1.1 ^ab^	6.9 ± 0.1 ^a^	8.1 ± 0.7

^(1)^ SSS: saturated-saturated-saturated; ^(2)^ SUS: saturated-unsaturated-saturated, Symmetric TAG; ^(3)^ SSU: saturated-saturated-unsaturated, Asymmetric TAG; ^(4)^ SUU: saturated-unsaturated-unsaturated, Asymmetric TAG; ^(5)^ USU: unsaturated-saturated-unsaturated, Symmetric TAG; ^(6)^ ND: not detected. All values are expressed as the mean ± standard deviation of the duplicated determinations. ^a–d^ Means with different letters along the same row among acyl migration reactants (0, 3, 24, and 100 h) are significantly different as determined by Duncan’s multiple range test at *p* < 0.05.

**Table 2:** Proposed triacylglycerol (TAG) molecular species of hydrogenated palm kernel oil (HPKO), shea butter, shea butter liquid, shea butter solid, refined acyl migration reactant (RAMR), and alternative fat analyzed by reversed-phase HPLC. (Unit: area%).

PN ^(1)^	Proposed TAG Species ^(2)^	HPKO	Shea Butter	Shea Butter Liquid	Shea Butter Solid	RAMR	Alternative Fat ^(4)^
24–34	CCLa/CLaC	12.2 ± 1.0	ND ^(3)^	ND	ND	ND	10.4 ± 0.8
	CLaLa/LaCLa						
	CaCaLa/CaLaCa						
	CaLaLa/LaCaLa						
	CaCaM/CaMCa						
36	LaLaLa	26.2 ± 4.7	ND	ND	ND	ND	27.8 ± 0.4
38	LaLaM/LaMLa	17.5 ± 2.1	ND	ND	ND	ND	12.3 ± 0.7
40–44	LaMM/MLaM	21.5 ± 0.1	ND	ND	ND	ND	11.2 ± 1.5
	LaLaP/LaPLa						
	LaMP/LaPM						
	MMM						
	LaPP/PLaP						
46	POL/OOL	4.3 ± 0.1	ND	ND	ND	ND	2.8 ± 0.0
	MOO/PPL						
	MPO						
	MPP						
48	OOO	ND	5.4 ± 1.9	10.5 ± 0.9	ND	8.5 ± 0.4	2.3 ± 0.1
	POO/OPO						
	POP/PPO						
	PPP	6.6 ± 1.9					3.6 ± 0.1
50	SOO/OSO	ND	32.0 ± 1.2	82.8 ± 2.0	2.6 ± 0.1	30.8 ± 1.3	9.1 ± 1.1
	POS/PSO	ND	5.9 ± 1.4	2.3 ± 1.2	4.0 ± 0.1	7.8 ± 0.2	1.6 ± 0.2
	PPS/PSP	4.2 ± 2.5	ND	ND	ND	ND	1.5 ± 0.2
52	SOS/SSO	ND	55.5 ± 4.8	4.3 ± 0.2	92.7 ± 0.4	51.2 ± 1.1	14.1 ± 1.5
	PSS/SPS	4.2 ± 2.5	ND	ND	ND	ND	1.0 ± 0.0
54	SOA/SAO	ND	1.1 ± 0.4	ND	1.3 ± 0.1	1.7 ± 0.3	0.9 ± 0.6
	SSS						
56	SSA/SAS	4.4 ± 2.3	ND	ND	ND	ND	1.8 ± 0.4

^(1)^ PN: partition number = total carbon number (CN)− 2× total number of double bonds (DB). ^(2)^ Fatty acid abbreviation of the proposed TAG species: C = caprylic acid (C8:0), Ca = capric acid (C10:0), La= lauric acid (C12:0), M = myristic acid (C14:0), P = palmitic acid (C16:0), S = stearic acid (C18:0), O = oleic acid (C18:1), A = arachidic acid (C20:0). ^(3)^ ND: not detected. ^(4)^ Alternative fat product was prepared by blending the RAMR and HPKO (4:6 blend, *w*/*w*).

**Table 3:** Fatty acid composition, slip melting point (SM), and complete melting point (CM) of whipping cream fat, hydrogenated palm kernel oil (HPKO), shea butter solid, refined acyl migration reactant (RAMR), and alternative fat. (Unit: %.)

Fatty Acids	Whipping Cream Fat	HPKO	Shea Butter Solid	RAMR	Alternative Fat ^(8)^
C4:0	0.17 ± 0.02	ND ^(1)^	ND	ND	ND
C6:0	0.44 ± 0.01	0.23 ± 0.01	ND	ND	0.14 ± 0.00
C8:0	2.58 ± 0.01	3.80 ± 0.03	ND	ND	2.25 ± 0.02
C10:0	3.21 ± 0.02	3.53 ± 0.01	ND	ND	2.11 ± 0.01
C12:0	34.44 ± 0.10	46.50 ± 0.01	ND	ND	27.78 ± 0.05
C14:0	14.93 ± 0.02	16.69 ± 0.01	ND	ND	9.94 ± 0.01
C14:1	0.34 ± 0.01	ND	ND	ND	ND
C16:0	16.56 ± 0.08	8.77 ± 0.01	3.53 ± 0.00	3.31 ± 0.00	6.57 ± 0.01
C16:1	0.52 ± 0.00	ND	ND	0.02 ± 0.00	0.01 ± 0.00
C18:0	19.10 ± 0.11	19.99 ± 0.01	55.80 ± 0.01	44.28 ± 0.05	29.82 ± 0.06
C18:1 *trans*	0.53 ± 0.03	0.17 ± 0.03	0.02 ± 0.00	0.05 ± 0.00	0.10 ± 0.02
C18:1 (*n*-9)	5.96 ± 0.05	0.09 ± 0.00	34.37 ± 0.00	45.04 ± 0.06	18.19 ± 0.02
C18:1 (*n*-7)	0.19 ± 0.04	ND	0.17 ± 0.00	0.07 ± 0.00	0.03 ± 0.00
C18:2 *trans*	0.23 ± 0.01	ND	0.14 ± 0.01	0.17 ± 0.00	0.06 ± 0.01
C18:2 (*n*-6)	0.55 ± 0.06	0.01 ± 0.00	4.02 ± 0.00	5.43 ± 0.00	2.19 ± 0.00
C20:0	0.22 ± 0.00	0.22 ± 0.00	1.76 ± 0.01	1.48 ± 0.00	0.73 ± 0.00
C20:1	ND	ND	0.12 ± 0.00	0.08 ± 0.12	0.07 ± 0.00
C18:3 (*n*-3)	0.11 ± 0.00	ND	0.06 ± 0.01	0.07 ± 0.00	0.02 ± 0.02
∑SFA ^(2)^	91.62 ± 0.08	99.73 ± 0.02	61.09 ± 0.01	49.06 ± 0.04	79.33 ± 0.00
∑USFA ^(3)^	7.59 ± 0.04	0.10 ± 0.00	38.75 ± 0.00	50.71 ± 0.05	20.51 ± 0.03
∑TFA ^(4)^	0.76 ± 0.02	0.17 ± 0.03	0.16 ± 0.01	0.22 ± 0.01	0.16 ± 0.03
AI ^(5)^	14.59	1220.3	0.09	0.07	3.61
SM (°C) ^(6)^	37.5 ± 0.7	39.3 ± 0.4	34.3 ± 0.4	28.5 ± 0.7	37.5 ± 0.7
CM (°C) ^(7)^	39 ± 0.0	40.3 ± 0.4	38.5 ± 0.7	41.5 ± 0.7	39.3 ± 0.4

^(1)^ ND = not detected; ^(2)^ ∑SFA = total saturated fatty acids (g/100 g); ^(3)^ ∑USFA = total unsaturated fatty acids (g/100 g); ^(4)^ ∑TFA = total trans fatty acids (g/100 g); ^(5)^ AI (atherogenicity index) = [(C12:0%) + 4 × (C14:0%) + (C16:0%)]/(∑USFA%); ^(6)^ SM = slip melting point; ^(7)^ CM = complete melting point; ^(8)^ Alternative fat was prepared by blending (RAMR:HPKO = 4:6, wt%) of refined acyl migration reactant (RAMR) and HPKO.

**Table 4:** Polymorphic crystal form and short spacings (Å) of hydrogenated palm kernel oil (HPKO) and alternative fat.

-	Short Spacings (Å)			-	-
	4.6	4.2	3.8	Relative Content ^(1)^ (β’:β)	Polymorphic Form
HPKO	ND	4.219	3.8301	100:0	β’
Alternative fat ^(2)^	4.6526	4.2543	3.8151	77:23	β’ > β

^(1)^ Relative contents of β’and β in crystal form were obtained from each intensity; ^(2)^ Alternative fat was prepared by blending (RAMR:HPKO = 4:6, wt%) of the refined acyl migration reactant (RAMR) and HPKO.

**Table 5:** Ingredients and their weight ratio (%) of the O/W emulsions for the preparation of whipping cream.

Ingredients		Ratio (% *w*/*w*)
Oil phase	Fat	25
	Lactylated monoglyceride (LMS)	0.2
	Glyceryl monostearate (MAG, C18:0)	0.5
Aqueous phase	Corn syrup	36
	Sodium caseinate	0.3
	Methyl cellulose	0.5
	Sodium stearoyl lactylate (SSL)	0.2
	Xanthan gum	0.1
	Distilled water (DW)	37.2

## Data Availability

All relevant data are included in the article.

## Sample Availability

Samples of the compounds are not available from the authors.
